# Using Different Surface Energy Models to Assess the Interactions between Antiviral Coating Films and phi6 Model Virus

**DOI:** 10.3390/jfb14040232

**Published:** 2023-04-19

**Authors:** Zdenka Peršin Fratnik, Olivija Plohl, Vanja Kokol, Lidija Fras Zemljič

**Affiliations:** Institute of Engineering Materials and Design, Faculty of Mechanical Engineering, University of Maribor, Smetanova ul. 17, SI-2000 Maribor, Slovenia

**Keywords:** films, surface free energy, SFE mathematical models, phi6, wettability, spreading, interactions

## Abstract

High molecular weight chitosan (HMWCh), quaternised cellulose nanofibrils (qCNF), and their mixture showed antiviral potential in liquid phase, while this effect decreased when applied to facial masks, as studied in our recent work. To gain more insight into material antiviral activity, spin-coated thin films were prepared from each suspension (HMWCh, qCNF) and their mixture with a 1:1 ratio. To understand their mechanism of action, the interactions between these model films with various polar and nonpolar liquids and bacteriophage phi6 (in liquid phase) as a viral surrogate were studied. Surface free energy (SFE) estimates were used as a tool to evaluate the potential adhesion of different polar liquid phases to these films by contact angle measurements (CA) using the sessile drop method. The Fowkes, Owens–Wendt–Rabel–Kealble (OWRK), Wu, and van Oss–Chaudhury–Good (vOGC) mathematical models were used to estimate surface free energy and its polar and dispersive contributions, as well as the Lewis acid and Lewis base contributions. In addition, the surface tension SFT of liquids was also determined. The adhesion and cohesion forces in wetting processes were also observed. The estimated SFE of spin-coated films varied between mathematical models (26–31 mJ/m^2^) depending on the polarity of the solvents tested, but the correlation between models clearly indicated a significant dominance of the dispersion components that hinder wettability. The poor wettability was also supported by the fact that the cohesive forces in the liquid phase were stronger than the adhesion to the contact surface. In addition, the dispersive (hydrophobic) component dominated in the phi6 dispersion, and since this was also the case in the spin-coated films, it can be assumed that weak physical van der Waals forces (dispersion forces) and hydrophobic interactions occurred between phi6 and the polysaccharide films, resulting in the virus not being in sufficient contact with the tested material during antiviral testing of the material to be inactivated by the active coatings of the polysaccharides used. Regarding the contact killing mechanism, this is a disadvantage that can be overcome by changing the previous material surface (activation). In this way, HMWCh, qCNF, and their mixture can attach to the material surface with better adhesion, thickness, and different shape and orientation, resulting in a more dominant polar fraction of SFE and thus enabling the interactions within the polar part of phi6 dispersion.

## 1. Introduction

Insight into interfacial interactions between different agents can be performed using various techniques, including XPS [1], AFM [2], and others [3]. Among them, the use of surface free energy (SFE) seems to be an attractive technique for understanding different materials’ behaviour when coming in contact due to its simplicity and use of several theoretical models to predict behaviour at interface. The latter has rarely been reported [4], if ever, when used for solid–liquid interactions between a model virus and biopolymer film surface.

In general, the SFE is a relative measure of excess energy on the material surface and correlates with the strength of bulk interactions (i.e., SFE is higher when bulk interactions are stronger or if surface exposure is greater). Adsorption, wetting, and adhesion of solid material with surrounding liquids depend on the SFE of the solid material and the surface free tension (SFT) of the liquid phase. Wetting of the material thus refers to the adhesion of a liquid phase to be spread out on a solid surface by the formation of adhesive bonds, the occurrence of adsorption, and the interaction between dispersion or polar forces [5]. The magnitude of the forces responsible for adhesion (weaker van der Waals and preferably stronger acid–base forces) depends mainly on thermodynamic quantities such as surface energy [6,7].

Additionally, surface energy plays a crucial role in the real system such as in the contact between bulk materials and biological organisms. Surface wettability is the driving force behind solid–liquid interactions in biological systems. SFE and wetting results from studies on interactions between different surfaces and bio-organisms have been helpful in understanding the phenomenon of adhesion [8]. For example, it is used to understand the interactions between microorganisms and different adsorbent surfaces, leading to a fouling effect and reduction in protein binding [9]; *Staphylococcus epidermidis* and the metal surface [10]; low-density polyethylene grafted with ascorbic acid, *S. aureus* and *E. coli bacteria* [11]; dental implants from zirconia (ZrO_2_) and titanium (Ti) and *F. nucleatum, P. gingivalis*, and *S. sanguinis* bacteria [12]; and adhesion between *Streptococcus mutans* and *Streptococcus mitis* and different bulk-fill resin composites used in dental treatment [13].

Obviously, the state of research has therefore shown that the SFE method can be very useful in better understanding material interactions between microorganisms. In most cases, the emphasis is on material interactions between bacteria and less b between viruses. As a useful tool for gaining insight into the mechanism of action of material surfaces with microorganisms, our hypothesis was that the SFE estimation can be successfully applied to understand some challenges from our previous research in the COVID-19 field relating to the preventive pillar, i.e., the development of novel personal protective materials, in which polysaccharide-based liquid formulations of chitosan, quaternised cellulose nanofibrils and their blends were prepared as liquid formulations that showed antiviral activity against the model virus phi6, which is commonly used as a surrogate for SARS-CoV-2 [14].

Chitosan [15,16,17,18,19,20], quaternised cellulose nanofibrils [21,22,23], and their blends were used to study interfacial interactions using a model virus, since they are already known for their excellent antibacterial activity, and, in recent years, they have also gained interest as antiviral agents, with gaps mainly in the understanding of interaction phenomena between these agents and specific viruses [24,25,26,27,28,29,30,31,32,33]. Mixtures of the two have also not been studied yet. According to excellent antiviral results (using bacteriophage phi6) for those components in the form of liquid formulations shown in our previous research [14], they were further applied by screen printing onto a polypropylene (PP) layer, which built an integral part of the protective mask [34]. To understand the antiviral activity of coatings when applied to material, we went deeper into understanding of the interaction phenomena using an SFE tool. Therefore, the goal was to gain insight into the adhesiveness of the virus phi6 to different biopolymer surfaces. It is important to know that there are great differences in the conformation, structure, and, consequently, chemistry (accessibility of groups) of polymers and nanofibrils when they are in the form of solution or are applied on a surface as a coating. In solution, the conformation depends on the pH while applied on a surface; macromolecules usually come in the form of a film with specific thickness and morphology, conformation and, consequently, lower accessibility to the surface functional groups responsible for antiviral activity due their possible interactions with the material’s surface. To this end, in order to elucidate the interactions between the virus and these biopolymer-coated surfaces, model films were prepared using spin-coating and analysed for SFE with the associated dispersion and polar contributions, and the spreading parameter was applied. SFE cannot be measured directly; thus, the most common evaluation method using CA measurements between film samples and different polar test liquids was required [35]. For determining the antiviral efficiency and viral mode of action with an antiviral biopolymer surface, the bacteriophage phi6 was applied (as already mentioned), as commonly used when investigating high-transmission coronaviruses since it is known as a no-risk viral surrogate for SARS-CoV-2 [36,37].

In following paper, CA results were used with more common mathematical methods, i.e., Fowkes, Owens–Wendt–Rabel–Kealble (OWRK), Wu, and van Oss–Chaudhury–Good (vOGC), providing a reliable estimation of the total SFE of prepared films and their associated dispersion and polar contributions, with additional correlations between different models used. The interactions between the SFE of solid samples and the SFT of different polar test solvents, and, in particular, their dispersion and polar contributions to wetting were also investigated. In addition, the spreading parameter, estimating possible correlations between the different polysaccharide films and different polar test solvents and for phi6 model virus dispersion in particular, was determined.

Providing the experimentally defined solid–liquid interactions using the concept of SFE and identifying its possible influence on wettability and spreading contribute to a better understanding of the interaction mechanisms between antiviral coatings and the model virus phi6, which has not been yet investigated. Therefore, knowledge of surface properties, including wetting, where a balance between adhesion and cohesion forces controls fluid spread on a surface, is an important contribution to the research area for the development of antiviral surfaces that are most effective against a particular type of virus. Understanding these types of interactions can help manipulate the coating so that it is as efficient as possible (in terms of high antiviral inhibition).

## 2. Materials and Methods

### 2.1. Materials

High molecular weight chitosan (HMWCh, 310–375 kDa, >75% deacetylated, supplied by Sigma-Aldrich, Vienna, Austria) solution was prepared using a weight concentration of 20 g/L. An appropriate amount of chitosan (powder) was weighed and suspended in Milli-Q ultrapure water (Millipore Direct 8; Labena, Slovenia). Under constant stirring, using a propeller stirring element (IKA Eurostar 20 high speed digital; Merck KGaA, Darmstadt, Germany), the pH of the solution was adjusted to pH 4 with the addition of concentrated acetic acid (99–100%; Sigma-Aldrich Merck KGaA, Darmstadt, Germany). The prepared solution was stirred at 500 rpm for 24 h at room temperature.

Water-suspended quaternised cellulose nanofibrils (qCNF) consisting of few μm long and up to 50 nm tight fibrils with around 0.23 degrees of substitution (DS) were prepared by Xylocel Oy, Finland, according to their patent WO/2016/075370 using glycidyltrimethylammonium chloride.

The mixture of HMWCh and qCNF at a 1:1 weight ratio (*w*/*w*) was also prepared using constant propeller stirring (IKA Eurostar 20 high speed digital; Merck KGaA, Darmstadt, Germany) at 500 rpm at night under ambient conditions.

A microscope slide cover glass (hard circular, fi = 14 mm; Glaswarenfabrik Karl Hecht GmbH & Co KG, Sonheim, Germany) was utilized as a substrate in a spin coating device to produce films using polymer formulations.

A 4 × SM buffer solution (SMb) (KFR) was prepared using 23.2 g of NaCl (KGaA, Darmstadt, Germany) and 8 g of MgSO_4_ × 7H_2_O (KGaA, Darmstadt, Germany) dispersed in 800 mL of Milli-Q ultrapure water. The 200 mL of Tris-HCl (1 M, pH 7.5; Sigma-Aldrich) and 20 mL 2% gelatine (Sigma-Aldrich) was added, and Milli-Q ultrapure water was filled to 1000 mL. The solution thus prepared was autoclaved at 121 °C for 15 min and stored at room temperature for further use. Then, a 1 × SM buffer was used as diluent and as storage buffer for phi6 phages. Thus, bacteriophage phi 6 (DSM 21518) with an initial concentration of 2 × 10^11^ PFU/mL was diluted 100× in 1× SM buffer.

The test liquids used in procedure for evaluating SFE, utilizing different calculation models, were diiodomethane (DI; 99% purity; Sigma Aldrich Merck KGaA, Darmstadt, Germany), dimethyl sulfoxide (DMSO; 99.9% purity; Honeywell Riedel-de Haën, Seelze, Germany), ethylene glycol (EG; 99% purity; Fisher Scientific Vienna, Austria), glycerol (GY; 88% purity; Alkaloid AD, Skopje, Macedonian), and Milli-Q ultrapure water (W; Millipore Direct 8; Labena, Slovenia). The test liquids were used as received.

In addition, the dispersion of a model virus phi6 (phi6) and SM buffer solution (SMb), the latter used as base solution for preparing a dispersion containing a phi6 model virus, were also used as test liquids in evaluating for solid–liquid interactions.

### 2.2. Polymer Formulation Characterisation

Among several parameters, such as the shape of the collecting substrate, centrifugal force, evaporation, SFT, and the viscosity of the liquid sample, could affect the uniformity of the formed films. Thus, the polymer formulations were used prior to produce films; they were additionally characterised.

#### 2.2.1. Surface Tension

The SFT of the polymer formulation was determined using a Wilhelmy plate (Pt plate) of known dimensions according to DIN 53 914 and a K12 tensiometer (KRÜSS GmbH; Hamburg, Germany). The Wilhelmy Pt plate was fixed in a special holder in the apparatus. A glass container filled with a sample liquid was placed within a movable table at the bottom of the apparatus. At the moment the tensiometer was switched on, the moving table raised the liquid sample until it came into contact with the bottom edge of the Pt plate. After wetting the bottom edge of the Pt plate, the moving table started to descend, and just before the plate came off, the surface of the polymer formulation—in terms of force proportional to the weight of the liquid sample suspended below the plate—was measured. The measurements were performed in triplicate for each polymer formulation.

#### 2.2.2. Viscosity

The viscosity measurements were performed using a Fungilab rotational viscometer (model Smart series, Barcelona, Spain) at room temperature. The polymer solution was poured into a sample holder and placed within the apparatus. The appropriate spindle was chosen (model TL) and fixed to the spindle support above the sample holder filled with polymer solution. Switching on the apparatus, the spindle stared to rotate, and after a definite time (2 min), the display recorded the measured value. Each polymer solution was measured three times.

### 2.3. Preparation of Spin-Coated Films

The spin coater (Polos, SPS Europe, Putten, The Netherlands) was used with a deposition technique followed by four key stages for generating homogeneous film samples. Firstly, the circular microscope slide cover glass was placed onto a vacuum holder, allowing for fixing (90–150 mbar) of the cover glass whilst distributing the liquid sample onto it after turning on the vacuum pump. By closing the lid, the preprogramed spin process was selected and then initiated. Secondly, the same quantity (0.4 mL) of a sample, using a syringe (5 mL, Luer-Lock, Chirana, Slovak republic), was put on the glass slide. During the rotation of the glass slide, the sample liquid formulation on the slide cover glass was spread. By the third step, the cover glass reached the desired rotation speed of 4000 rpm/s with accel/decel = 2500 rpm/s during the 30 s procedure time. During this stage, the entire the sample formulation became level. In the final stage, the evaporation of the sample formulation dominated, resulting in a formed film. The sample notation of the produced films using natural-based formulations along with descriptions is listed in Table 1.

### 2.4. Weight Uniformity and Thickness Evaluation of Film Samples

Weight of the spin-coated films was determined upon gravimetric evaluation using a digital balance (Mettler Toledo International Inc, Columbus, OH, USA). Thickness of the films was measured at three different randomly selected positions using a digital vernier calliper (Fisher Scientific International Inc., Waltham, MA, USA). The measurements were repeated three times for each sample and a mean value was calculated along with standard deviation. The used samples were dried and conditioned.

### 2.5. Optical Microscopy of the Spin-Coated Films

The film formation as well as the morphology of spin-coated samples with biopolymers were observed with a Kern optical light microscope using an ODC 825 camera at 400× magnification. At the same time, the microscope was connected to a computer via USB to observe the image on the screen and take photos.

### 2.6. Wettability Assessment

Surface properties of a solid material influence the adhesion of biological molecules; thus, wettability is among the important parameters emphasising the latter. To predict the wetting behaviour of materials brought into contact, SFE evaluation is used. The first step in evaluation is to choose the proper set of test liquid phases (regarding their polar and dispersive components of the SFT) matching the calculation model applied, followed by CA measurements.

#### 2.6.1. Surface Tension, Polar, and Dispersive Components of Liquid Forms

Firstly, the SFT of test liquids (i.e., diiodomethane (DI), dimethyl sulfoxide (DMSO), ethylene glycol (EG), glycerol (GY), and Milli-Q ultrapure water (W)), as well as model virus phi6 (phi6) dispersion and SM buffer (SMb) solution were determined using the Wilhelmy plate method (DIN 53 914) on a Processor Tensiometer K12 (Krüss GmbH, Hamburg, Germany). The polar ($\gamma_{l}^{P}$) and dispersive ($\gamma_{l}^{D}$) SFT components of the liquid phases were evaluated upon CA measurements using a standard poly(tetrafluoroethylene) (PTFE) plate on the same device.

#### 2.6.2. CA Measurements with Liquid Forms

The glass cover sample coated with films using A, B, and AB formulations was placed on the table under a stainless-steel needle (0.16 mm inner diameter; DataPhysics Instruments GmbH, Filderstadt, Germany) within the goniometer (OCA 35 DataPhysics Instruments GmbH, Filderstadt, Germany). The liquid form (i.e., SMb, DI, DMSO, phi6, EG, GY, and W) was placed in a 500 μL glass tube (DataPhysics, Filderstadt, Germany) and automatically pushed further along a PTFE plastic tube (DataPhysics, Germany) resulting in the formation of a droplet. The sessile drop method was used for performing CA measurements. A droplet, 3 μL in volume, with a dosage rate of 0.50 μL/s, was dispensed onto the sample surface (i.e., glass cover sample with film A, B, AB, and phi6), thus forming the static CA. The result was collected, and the procedure was repeated at least 2–4 times on each glass cover (depending on size of film-coated area), while within each set of samples, up to 104 data points were collected.

### 2.7. Estimation of Surface Energy Using Different Models

Surface energy can be estimated using indirect CA measurements with different models. Most models are based on Young’s theory, combining the surface energy of the solid (*γ_s_*), SFT of liquids (*γ_l_*), and interfacial energy (*γ_sl_*) between them [38,39] as:

(1) $$\gamma_{s=\gamma_{sl}}+\gamma_{l}cos\varphi$$

where *φ* is the CA between the solid and test liquid.

Dupré’s equation describes interfacial relations and solid/liquid interactions. The latter are expressed as the work of adhesion (*W_sl_*) that must be carried out to separate solids and liquids or as energy released from wetting [38,39]. The relation is similar, but not the same as that between the interfacial tension and spreading coefficient (*S_p_*), as the following equation

(2) $$\gamma_{sl}=\gamma_{s}-\gamma_{l}-S_{p}$$

emphasises the liquids’ tendency to wet the solid. Young and Dupré equitation can be combined, forming a Young–Dupré equation, presenting the platform for surface energy calculation models as follows:

(3) $$W_{sl}=\gamma_{l}(1+\cos\varphi )$$

Four calculation models were chosen for estimating the antiviral films’ SFE. The Fowkes’ model is more often utilized for nonpolar polymers; thus, it was used for estimation of dispersive component interactions. The Owens–Wendt–Rabel–Kelble (OWRK) is one of more commonly used models for SFE evaluation, similar to the Fowkes model; it was used because it fits better to surfaces with lower energy. The Wu calculation model was applied since it is more frequently used to evaluate the SFE of organic polymers, as spin-coated films are, but it is not generally put to use. The van Oss–Chaudhury–Good (vOGC) calculation model is, among those listed, the newest and was also chosen to evaluate the acid and base components contributing to polar interactions.

#### 2.7.1. Fowkes Calculation Model

Fowkes proposed a model [40] where the surface energy of a solid/liquid is the sum of versatile components, capable of interactions; he mainly investigated two-phase systems (i.e., solid–liquid) where dispersion forces dominate. Focusing on the latter and using the geometric mean, the interfacial interactions could be expressed as follows:

(4) $$\gamma_{sl}=\gamma_{s}+\gamma_{l}-2\sqrt{\gamma_{s}^{d}\gamma_{l}^{d}}$$

The combination of Equation (4) and Young’s equation (Equation (1)), taking into account that $\gamma_{s}=\gamma_{s}^{d}$, leads to the following calculation:

(5) $$\gamma_{s}=\frac{\gamma_{l}^{2}{\left(1+\cos\varphi \right)}^{2}}{4\gamma_{l}^{d}}$$

The first step in evaluation of SFE is to measure the CA using the liquid possessing only a dispersive component (i.e., $\gamma_{l}^{d}$) and taking into account that $\gamma_{l}=\gamma_{l}^{d}$. Thus, the upper equation (Equation (5)) could be rewritten as

(6) $$\gamma_{s}=\gamma_{s}^{d}=\frac{1}{4}\gamma_{l}{\left(1+cos\varphi \right)}^{2}$$

which allows for obtaining the dispersive SFE ($\gamma_{s}^{d})$ part of the solid.

The next step is to measure the CA using a liquid with known dispersive and polar components, i.e., $\gamma_{l}=\gamma_{l}^{p}+\gamma_{l}^{d}$. Using Fowkes’ theory, i.e., where SFE of a solid is the sum of independent components, saying that, except $\gamma_{s}^{d}$, all other individual components can be linked to polar interactions $\gamma_{s}^{p}$, then the following equation is presented:

(7) $$\gamma_{sl}=\gamma_{s}+\gamma_{l}-2\sqrt{\gamma_{s}^{d}\gamma_{l}^{d}}-2\sqrt{\gamma_{s}^{p}\gamma_{l}^{p}}$$

This, along with the calculated $\gamma_{s}^{d}$ and measured CA with liquid possessing both polar and dispersive components, could be used to calculate the polar component of the solid surface energy as follows:

(8) $$\gamma_{s}^{p}=\frac{{\left(\frac{1}{2}\gamma_{l}\left(1+cos\varphi \right)-\sqrt{\gamma_{s}^{d}\gamma_{l}^{d}}\right)}^{2}}{\gamma_{l}^{p}}$$

#### 2.7.2. Owens–Wendt–Rabel–Kelble Calculation Model

Using the Owens–Wendt–Rabel–Kelble (OWRK) model [41,42], based on Fowkes’ theory (Equation (7)) and Young’s equation (Equation (1)), the OWRK equation could be written as:

(9) $$\sqrt{\gamma_{s}^{d}\gamma_{l}^{d}}+\sqrt{\gamma_{s}^{p}\gamma_{l}^{p}=}\frac{1}{2}\gamma_{l}(1+cos\varphi )$$

This is similar to Fowkes’ model but differs in the method of SFE calculation. It can be expressed as a slope intercept formula, i.e., *y = mx + c*, where

(10) $$y=\frac{\gamma_{l}(cos\varphi +1)}{2\sqrt{\gamma_{l}^{p}}};\;c=\sqrt{\gamma_{s}^{d}};\;m=\sqrt{\gamma_{s}^{p}}\;\mathrm{and}\;x=\frac{\sqrt{\gamma_{l}^{p}}}{\sqrt{\gamma_{l}^{d}}}$$

**Figure 1:**
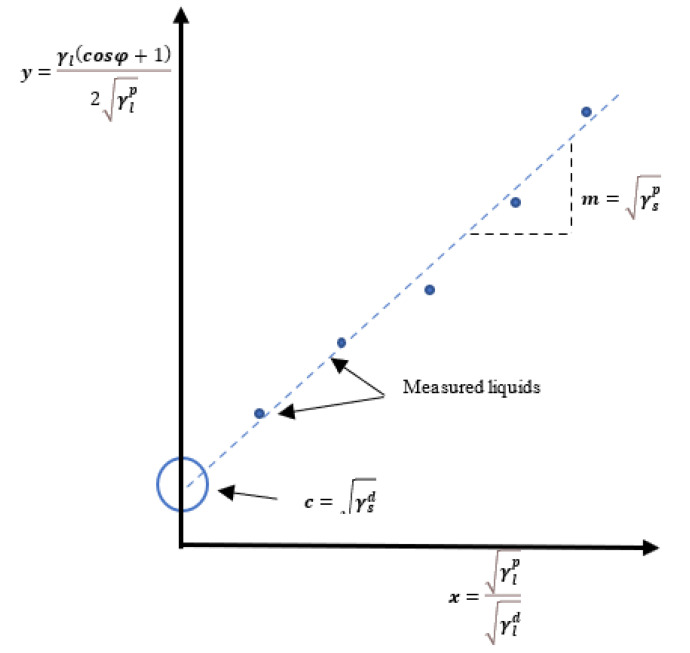
An OWRK plot, where CA is plotted against SFT and the components of surface energy are found from the intercept and gradient of a best fit line.

The liquid with a dominant polar component should be chosen as one of the test liquids and the liquid with dispersive dominant component as the other one. Performing CA measurements with test liquids where γ, $\gamma_{l}^{p}$, and $\gamma_{l}^{d}$ are known, the values *x* and *y* could be determined, as shown in Figure 1 (i.e., polar components $\gamma_{s}^{p}=m^{2}$ and dispersive component $\gamma_{s}^{d}=c^{2}$).

#### 2.7.3. Wu Calculation Model

Wu [43] followed the idea of OWRK and proposed an equation wherein harmonic instead of geometric means for interfacial interactions between a polymer and a liquid is taken into account:

(11) $$\gamma_{sl}=\gamma_{s}+\gamma_{l}-4(\frac{\gamma_{s}^{d}\gamma_{l}^{d}}{\gamma_{s}^{d}+\gamma_{l}^{d}}+\frac{\gamma_{s}^{p}\gamma_{l}^{p}}{\gamma_{s}^{p}+\gamma_{l}^{p}})$$

#### 2.7.4. Van Oss–Chaudhury–Good Calculation Model

The van Oss–Chaudhury–Good model (vOGC) [44,45] considers that dispersive and polar interactions are involved with many surfaces. Moreover, the model also considers interactions (i.e., hydrogen bonds) that were not taken into account by previous models. The surface energy is thus expressed as:

(12) $$\gamma_{s}=\gamma_{s}^{LW}+\gamma_{s}^{AB}\;\mathrm{where}\;\gamma_{s}^{AB}=2\sqrt{\gamma_{s}^{+}\gamma_{s}^{-}}$$

where $\gamma_{s}^{LW}$ is a Lifshitz–van der Waals component involving long-range interactions (i.e., London, Keesom and Debye); $\gamma_{s}^{AB}$ is the acid–base component concerning short-term acid–base interactions; $\gamma_{s}^{+}$ is the acid component explaining the ability of a surface to interact with a basic liquid (i.e., one that can donate electron density) through polar interactions, such as dipole–dipole bonding and hydrogen bonding; $\gamma_{s}^{-}$ is the base component involving the opposite of acid i.e., interactions with an electron-accepting, acidic liquid.

Based on interaction studies between biopolymer liquids and hydrophobic surfaces [45], the following relationship was derived:

(13) $$\gamma_{sl}={\left(\sqrt{\gamma_{s}^{LW}}-\sqrt{\gamma_{l}^{LW}}\right)}^{2}+2(\left(\sqrt{\gamma_{s}^{+}}-\sqrt{\gamma_{l}^{+}}\right)\left(\sqrt{\gamma_{s}^{-}}-\sqrt{\gamma_{l}^{-}}\right))$$

Bearing in mind the calculation $\gamma^{AB}=2\sqrt{\gamma^{+}\gamma^{-}}$ and Young’s equation (Equation (1)), the following relation could be obtained:

(14) $$\frac{1}{2}\gamma_{l}\left(1+cos\varphi \right)=\sqrt{\gamma_{s}^{LW}\gamma_{l}^{LW}}+\sqrt{\gamma_{s}^{+}\gamma_{l}^{-}}+\sqrt{\gamma_{s}^{-}\gamma_{l}^{+}}$$

Solving the unknown entities, i.e., $\gamma_{s}^{LW}$, $\gamma_{s}^{+}$, and $\gamma_{s}^{-}$, three test liquids must be used to measure CAs. First, liquid possessing only disperses a part, the CA can be used to calculate $\gamma_{s}^{d}$ (Equation (15)), continuing by measuring the CA with liquid with no acid component to calculate $\gamma_{s}^{+}$ (Equation (16)), and, finally, the CA using the liquid with only an acid component to define the $\gamma_{s}^{-}$ (Equation (17)):

(15) $$\frac{1}{2}\gamma_{l}\left(1+cos\varphi \right)=\sqrt{\gamma_{l}^{d}\gamma_{s}^{d}}$$

(16) $$\frac{1}{2}\gamma_{l}\left(1+cos\varphi \right)=\sqrt{\gamma_{l}^{d}\gamma_{s}^{d}}+\sqrt{\gamma_{l}^{-}\gamma_{s}^{+}}$$

(17) $$\frac{1}{2}\gamma_{l}\left(1+cos\varphi \right)=\sqrt{\gamma_{l}^{d}\gamma_{s}^{d}}+\sqrt{\gamma_{s}^{-}\gamma_{l}^{+}}$$

The overall surface energy of the solid is then calculated as a sum of all obtained values. Another approach is to use liquid with both acid and base component and since the $\gamma_{s}^{d}$ is already calculated along with $\gamma_{s}^{+}$ or $\gamma_{s}^{-}$, the remaining missing component could be calculated using Equation (14).

### 2.8. Solid–Liquid Phase Interactions

The deposition of a liquid phase onto a solid generates new interfaces between dissimilar materials and involves considerations of wettability, spreading, interface evolution, and adhesion. The wettability of a solid using a liquid is characterized in terms of the angle of contact that the liquid makes on the solid [46,47,48] and could be expressed as the relationship between the surface energy of a solid and SFT of a liquid using the relationship proposed by Grifalco and Good [49] by introducing the parameter *Φ*, which highlights the interfacial interactions:

(18) $$\gamma_{sl}=\gamma_{s}+\gamma_{l}-2\Theta \sqrt{\gamma_{s}\gamma_{l}}$$

In order to obtain the following relationship:

(19) $$cos\theta =2\Theta \sqrt{\frac{\gamma_{s}}{\gamma_{l}}}-1$$

The proposed relationship (Equation (19)) suggests that interfacial energy is proportional to *cos φ* and complete wetting occurs if *cos φ* = 1; thus, *Φ* would reach its maximum. However, *cos φ* did not perfectly correlate with *Φ*, since in solid-liquid interactions, the surface energy components of both involved parties must be considered. In addition, liquids with the same overall SFE could possess different SFE components resulting in different wetting behaviours [50]. The polar ratio of the liquid (*P_l_*) and solid (*P_s_*) reflects the wettability effect. The influence of surface energy components on wetting can be explained by combining Equations (7) and (18), resulting in

(20) $$\Theta \sqrt{\gamma_{s}\gamma_{l}}=\sqrt{\gamma_{s}^{d}\gamma_{l}^{d}}+\sqrt{\gamma_{s}^{p}\gamma_{l}^{p}}$$

(21) $$\Theta =\sqrt{\frac{\gamma_{s}^{d}\gamma_{l}^{d}}{\gamma_{s}\gamma_{l}}}+\sqrt{\frac{\gamma_{s}^{p}\gamma_{l}^{p}}{\gamma_{s}\gamma_{l}}}=\sqrt{\left(1-P_{s})(1-P_{l}\right)}+\sqrt{P_{s}P_{l}}$$

#### Spreading Coefficient

The work carried out on the interface may be defined as four key wetting (work) functions [51]:

(22) $$\mathrm{Cohesion}\;(W_{c})\;W_{c}=2\gamma_{l}orW_{c}={2\gamma }_{s}$$

(23) $$\mathrm{Adhesion}\;(W_{A})\;W_{A}=\gamma_{s}+\gamma_{l}-\gamma_{sl}=\gamma_{l}(cos\varphi +1)$$

(24) $$\mathrm{Immersion}\;(W_{i})\;W_{i}=\gamma_{s}-\gamma_{sl}=\gamma_{l}cos\varphi$$

(25) $$\mathrm{Spreading}\;(W_{s})\;W_{s}=\gamma_{s}-\left(\gamma_{sl}+\gamma_{l}\right)=\gamma_{l}(cos\varphi +1)$$

By combining Equations (7) and (25), the following relation is obtained for the spreading parameter (*S_p_*) as [52]:

(26) $$S_{p}=2(\sqrt{\gamma_{s}^{d}\gamma_{l}^{d}}+\sqrt{\gamma_{s}^{p}\gamma_{l}^{p}}-\gamma_{l})$$

The spreading parameter (*S_p_*) distinguishes the two different regimes of wetting, i.e., total wetting when *S_p_* > 0 in the case that the liquid completely wets the surface and lowers the surface energy. Partial wetting occurs when *φ* < 90°, denoted as mostly wetting, and *φ* > 90°, known as mostly nonwetting, indicating lower interactions between liquid and solid.

## 3. Results and Discussion

### 3.1. Thin Film Characteristics and Optical Microscopy of Spin-Coated Films

The SFT and viscosity of polymer formulations used prior in spin coating are shown in Table 2, since they are among the parameters influencing the film formation process. The weight and thickness results of the obtained spin-coated films on a glass cover using A, B, and AB sample formulations are also listed in Table 2, provided as mean values with standard deviations.

Instead of rectangular substrates, circular glass covers were used for film deposition using A, B, and AB formulations due to the circular vacuum holder within the spin coating apparatus. Moreover, the study reported by Yan et al. [53] proved that the shape of the substrate used is important and has less influence on thickness, since if the rectangular substrates were used, only up to 15% larger thickness could be obtained.

The comparison of results (Table 2) shows the weight variation depending on thickness of the formed films, i.e., the heaviest sample logically possess the largest thickness (film B) while the smallest thickness contributed to light weight when using sample AB.

The preliminary study showed that evaporation has a greater influence on thickness compared to substrate shape and procedure time. A higher rotation speed increases the air velocity on the surface for depositing the films, and results in better evaporation during film formation. Thus, using a speed of 4000 rpm, uniform films were obtained along the whole radius of the circular glass cover, since there was no evidence of higher thickness on the edge compared to middle (seen using optical microscopy, not shown here). The film started to dry at the edge and hindered the flow from middle toward edge, thus preventing an undesired accumulation. The evaporation was ongoing; thus, the thickness across the entire circular glass was uniform.

Solvent evaporation was the leading factor when using film AB, since its thickness was the smallest. The was also due to the polymer viscosity formulation used to form the aforementioned films, which was the lowest. The low formulation viscosity that formed film AB enhanced radial outflow; thus, more AB formulation could be evaporated during centrifugal force activity, resulting in the formation of a thinner film. The uniform thickness of prepared A and AB films was seen throughout the whole area on a glass cover (see standard deviation, Table 2), pointing out that the time used to spin both films enabled the formation of uniform films without any accumulation on any particular place within the substrate. The highest thickness was obtained using sample B, since the measured polymer viscosity formulation used to spin film B was also the highest. Moreover, the less uniform thickness using film made of polymer formulation B was due to fibres as seen randomly distributed on the cover slide, resulting in a higher standard deviation in terms of measured thickness (Table 2).

In addition, the SFT polymer formulation was lower for sample A and AB compared to sample B, indicating that SFT was smaller compared to centrifugal force. Regarding the latter, the decrease in cohesion force (low SFT) within the A and AB liquid sample resulted in greater wettability; thus, a rather uniform film was coated on the glass slide.

**Figure 2:**
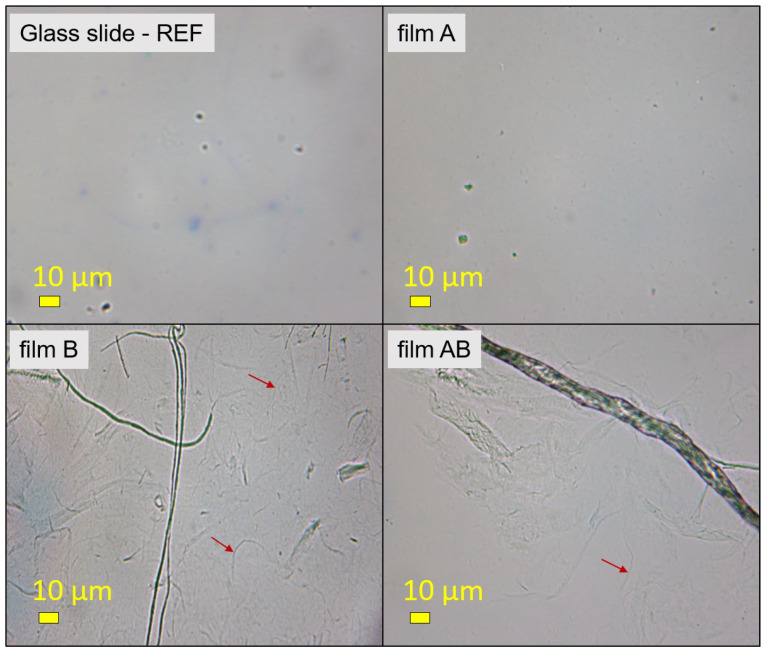
Optical microscopy images of a glass slide as reference and spin-coated films A, B, and AB at 400× magnification. Red arrows indicate qCNF nanofibers.

To show the uniform film formation and, at the same time, the morphology of the individual formulations (i.e., A and B) and their mixtures (i.e., AB) spin-coated onto glass slides, optical microscopy was used for all three dried films (Figure 2). A photograph of a pure glass slide was also taken for reference. Film A did not show any particular morphology in the form of a film; only some dark spots could be seen, possibly due to the fact that the HMWCh polymer was not completely dissolved. qCNF in the form of a film (such as B) showed larger fibres, which is consistent with the manufacturing process of qCNF, where larger microfibers are also present. Nevertheless, some nanofibers were also observed, marked with red arrows. In the case of the AB film, larger fibres of kat-CNF were observed with a smaller extension of the smaller nanofibers, which were probably covered with an A film. This again shows the dominant character of HMWCh in the AB formulation, as already highlighted in our previous article [34].

### 3.2. Surface Wetting

#### 3.2.1. SFT, Polar and Dispersive Components, and Polarity of Liquid Forms

The results of the measured SFT and its calculated polar and dispersive components of the test liquids used, applied to SFE calculation models, are listed in Table 3, along with the ratio of their polarity (*P_l_*). In addition, SM buffer solution (SMb) and viral surrogate dispersion (phi6) were also used in the liquids’ surface property evaluations to estimate their wettability potential regarding the film samples.

The measured values, as listed in Table 3, are in accordance with previously published data [54,55,56]. The lowest SFT was evident using DMSO, the highest using Milli-Q ultrapure water, while the SMb (buffer solution) and phi6 model virus dispersion exhibited 60.5 mN/m and 51.9 mN/m, respectively. The smallest contribution of polar components was seen in the SMb solution, reflected as the smallest polarity, followed by diiodomethane (DI), dimethyl sulfoxide (DMSO), model virus dispersion (phi6), ethylene glycol (EG), and glycerol (GY), while the biggest polarity was displayed using Milli-Q ultrapure water (W). Using the phi6 dispersion, the dispersive component dominated over the polar one, but approx. 29% of whole SFT was still devoted to polarity.

#### 3.2.2. CA Results Using Liquid Samples

Using a static method, a droplet of the test liquid, buffer solution, and viral surrogate dispersion with defined SFT properties (Table 3) were deposited onto spin-coated film samples A, B, and AB. By looking the shape of the drop, the CA was measured and the results of the average values, including standard deviation (SD), are reported in Table 4. The CA results obtained on film A, B, and AB were compared to results measured on the pure glass slide sample (used as a reference (REF)). In addition, the CA with Milli-Q ultrapure water was measured on spin-coated film using phi6 dispersion to additionally evaluate the model virus film’s hydrophilic/hydrophobic properties.

Using the test liquids diiodomethane (DI), dimethyl sulfoxide (DMSO), ethylene glycol (EG), glycerol (GY), and SMb solution, the highest CA was obtained using the reference sample (REF). The sample coated with film A and sample coated with AB followed and showed almost the same value (see Table 4). The smallest measured CA for all these tested liquids with the exception of phi6 was obtained from the sample covered using film B.

The opposite trend was observed in all film samples when depositing Milli-Q ultrapure water (W) as test liquid and phi6 model virus dispersion. Namely, the reference sample (REF) revealed the smallest measured value of CA, i.e., 78.6° using W and 82.3° using phi6 dispersion. The AB film followed with 90.1° when using phi6 dispersion and 103° by W. The sample coated with film A showed the highest CA value using W as the test liquid (117.7°) and using phi6 dispersion (98.9°).

The high CA value indicates fewer interactions between two liquid–solid phases being in contact, meaning that the cohesive forces were stronger than the adhesive forces; thus, the liquid phase molecules tended to rather interact between themselves than with the solid surface. The polar liquids (see Table 3, polarity) form large CAs on nonpolar solids, while liquids with no polar part form small CAs on nonpolar solids, i.e., they exhibit equal interaction. According to the CA results in Table 4, Milli Q ultrapure water (W) was the most polar (i.e., polarity of 0.7, see Table 3) [57] among all liquid phases used, forming large CA on nonpolar solids; thus, one could assume that film A and film AB could indirectly be estimated as more nonpolar and are obviously more favourable for dispersion interactions. On contrary, the reference sample (REF) formed the smallest CA with water and the largest with SMb (polarity of 0.02, see Table 3), indicating that, on the glass slide, the polar components are greater than those dispersed. Film B showed lower CA values compared to the other two films, and there was no apparent correlation between CA and the polarity of the solvents chosen. In fact, film B formed the same CA with both the solvent of lowest polarity (SMb) and the solvent of highest polarity (W). However, due to the good wettability with DMSO, it could also be predicted that dispersion interactions were present. However, it must be kept in mind that the spin-coating procedure also yields the highest weight and thickness of B-film and, crucially, the B-film samples within the set are likely to, consequently, have different conformations and associated accessibility of functional groups for interactions with the test solvent.

#### 3.2.3. Surface Energy of Polysaccharide-Based Film as Simulation of Thin Film Coatings 

**Figure 3:**
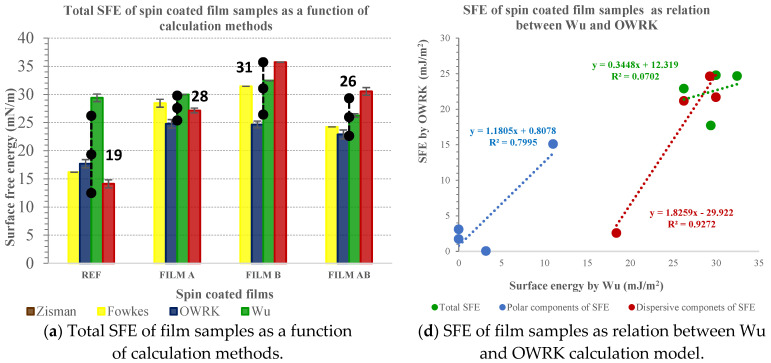

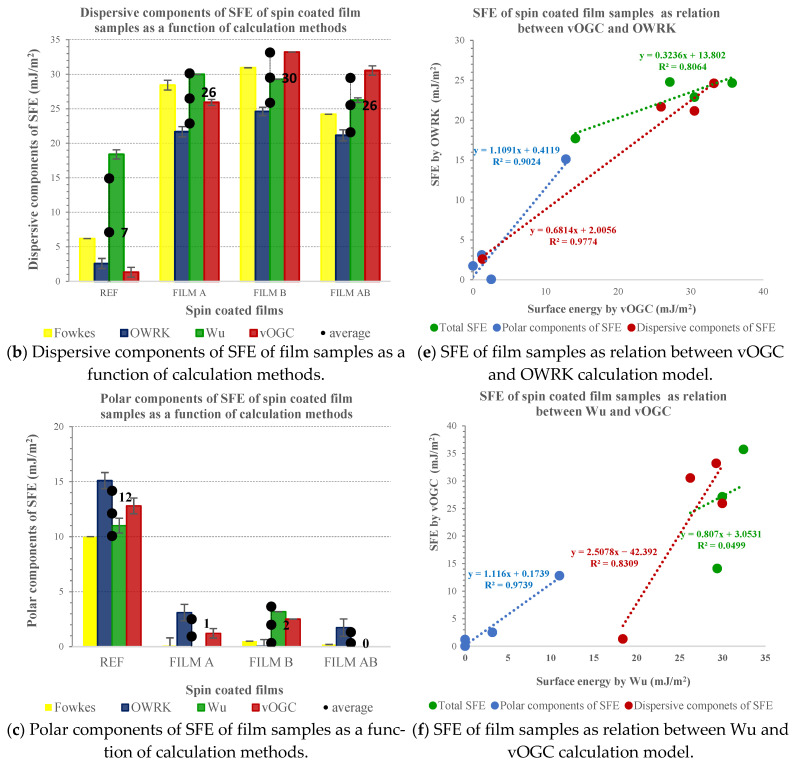
Total surface energy (**a**), dispersive components (**b**), and polar components (**c**) of the total SFE of reference and spin-coated films A, B, and AB on glass slide as a function of calculation models and the relationship between models for calculation total SFE, dispersive and polar components of the SFE, i.e., Wu vs. OWRK (**d**), vOGC vs. OWRK (**e**), and Wu vs. vOGC (**f**).

The estimated values of surface energy and their corresponding components of polysaccharide films were defined using different calculation models. The total surface free energy (SFE) in Figure 3a, the dispersive components in Figure 3b, and the polar components of SFE in Figure 3c of spin-coated polysaccharide films A, B, and AB on a glass slide, compared to the reference sample, are presented as a function of the three different calculation models chosen, i.e., Fowkes, Owens–Wendt–Rabel–Kaeble (OWRK), Wu, and van Oss–Chaudhury–Good (vOGC). The results are presented as obtained by each model along with average values and standard deviations.

In addition, Figure 3d–f presents the relationship between different models for evaluating the SFE and their components used; the relation between OWRK and Wu (Figure 3d), the relation between OWRK and vOGC (Figure 3e), and the relation between vOGC and Wu (Figure 3f) calculations of total SFE and their dispersive and polar components of spin-coated films A, B, AB, and the reference.

The highest average total SFE (Figure 3a) was evaluated as film B (31 mJ/m^2^), followed by film A (28 mJ/m^2^) and AB (26 mJ/m^2^). In all film samples, the SFE consisted mostly of contribution of due dispersive (see Figure 3b) components (>93%), while polar contribution (Figure 2c) was insignificantly low, i.e., from zero for sample AB, 1 mJ/m^2^ for sample A and up to 2 mJ/m^2^ for sample B. The lowest total SFE was estimated in the reference sample (REF), i.e., 19 mJ/m^2^, where the contribution of dispersive components to the total SFE was low, i.e., 7 mJ/m^2^, while the polar contribution was the highest (i.e., 12 mJ/m^2^). The latter is of base component origin (12 mJ/m^2^), contributing merely to the polar components of the REF sample.

The propensity of film A’s surface was indicated to interact in an acidic manner to the chosen liquid phases, since the acid component contributing to the polar component amounted to 1.21 mJ/m^2^ (not shown here). The surface of film B contained both acid (0.51 mJ/m^2^) and base sites, while the latter dominated (2 mJ/m^2^) (not shown here). The surface of film AB showed no acid–base properties. The latter may be explained by the basic and acid component interactions, thus not being available as individual sites. To sum up, the surface of glass covers coated with films A, B, and AB consists of mostly main chain length hydrocarbons of polysaccharide backbones, which could thus explain the large contribution of dispersed components compared to the polar contribution to total SFE (Figure 3). Obviously, the orientation of the polar group may slide toward the inner part of the glass, whilst hydrocarbon chains are oriented towards the surface. The nanocellulose (film B) seemed to be relatively more composed of electron donor material ($\gamma_{s}^{-}\gg \gamma_{s}^{+}$) even though the calculated base component using the vOGC model was small, i.e., 2 mJ/m^2^, but it was still higher compared to film A and AB. Thus, the present -OH and -COOH groups in nanocellulose (film B) as determined previously using XPS [34] act as a Lewis base, donating a pair of nonbonding electrons with the liquid phase in contact. It should be noted that quaternisation of nanocellulose introduces NH4^+^, which is one of the Lewis acids. Chitosan (film A) showed strong cationic character as it was dissolved in acidic solution, which led to protonation of the amino groups, and protons are known to be a good Lewis acid [58].

The R-square was used as a good-to-fit measure for linear regression analysis when comparing OWRK and Wu as two-component models to the three-component model (vOGC) in evaluating total SFE and their corresponding dispersed and polar contributions. Only the relation between vOGC and OWRK models showed noteworthy association in all three categories, i.e., total SFE and their corresponding compounds, i.e., a significant R-coefficient for the polar (R^2^ = 0.90) and even more meaningful for the dispersive (R^2^ = 0.97) component while showing moderate association (i.e., 0.80) for total SFE. The Wu model only showed good correlation (R^2^ = 0.92) with OWRK for dispersed and with vOGC (R^2^ = 0.97) for polar components. The reason for the good association between vOGC and OWRK was due to more than three liquid phases being used to obtain the CA results further applied in estimation of the SFE. The different model evaluations of the SFE of a solid surface by means of CA measurements may depend in whole or only in part on the set and number of liquid phases used [59].

**Figure 4:**
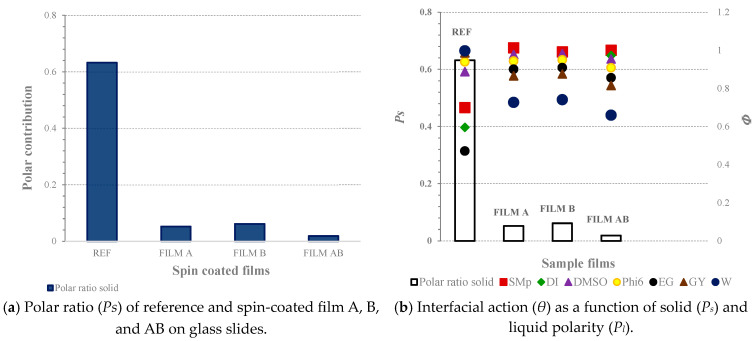
The polar contribution (*P_s_*) to the total SFE of reference and spin-coated films A, B, and AB on glass slides (**a**), and the interfacial action (*Φ*) as a function of solid (*P_s_*) and liquid polarity (*P_l_*) (**b**).

In Figure 4a, the polar ratio (*P_s_*) of the reference and spin-coated films A, B, and AB on glass slides calculated according to the total SFE, and in Figure 4b, the interfacial action (*Φ*) as a function of solid (*P_s_*) and liquid polarity (*P_l_*), are presented.

The surface energy of the solid sample could be reflected as a polar ratio; thus, the *P_s_* of films A, B, and AB are presented in Figure 4a. For film AB, followed by film A and film B, the contribution of the polar component to the total SFE was very small, close to zero. The latter emphasis on dominant dispersive components using all spin-formed samples, as seen in Figure 3b, connected to the available hydrocarbon chains in polysaccharide backbones, as already mentioned. The reference sample (REF) showed the lowest total SFE (see Figure 3a) among all tested samples, while its polar contribution *(P_s_* in Figure 4a) was the highest. The reference sample, made of glass, consisted of oxygen (O) and silicon (Si) molecules, where each Si was covalently bound to two oxygen molecules. The oxygen was more electronegative, and thus had greater affinity for attracting electrons compared to Si. Evidently, the resulting difference in electronegativity thus makes the substrate more polar.

Figure 4b presents the influence of solid–liquid components on wetting. Maximum wetting occurred when *P_s_* = *P_l_*; thus, *Φ* is equal to 1. Considering this, the max *Φ* of 1 appears when *P_s_* values are the same as *P_l_*, i.e., 0.02, 0.04, 0.18, 0.29, 0.40, 0.47, and 0.70 for SMb, DI, DMSO, phi 6, EG, GY, and W, respectively (see Table 3, last column). Interfacial function *Φ* results are different, as they depended on the polarity of the wetted liquids used. In more polar liquids (i.e., GY, W), *Φ* only showed the max value at a certain polarity for the reference solid. For the reference solid, the max *Φ* appeared at 0.47 for GY and at 0.70 for W. The latter could be supported by lower CA values (see Table 4) for the reference sample obtained using the two mentioned liquids (i.e., W and GY) and its present polar part. Results of *Φ* as a function of the solid samples’ polarity indicates that for film A, B, and AB, there was no chance that the surface would be wetted by the chosen standard polar test liquids and solutions used. The latter is not surprising, since in all of the films, the dispersive contribution to SFE dominated; thus, in relation to Figure 4b, pointing out only the polarity, this is more clearly expressed.

#### 3.2.4. Influence of Surface Energy and Their Components on Wettability

**Figure 5:**
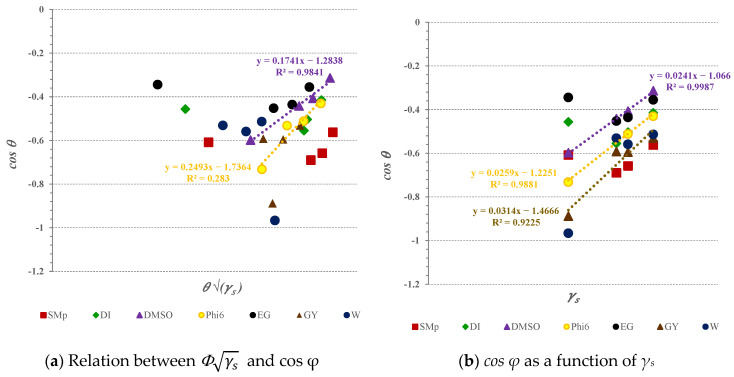
The interactions between solid samples (REF, films A, B, and AB coated on glass slides) and test liquid forms, presented as relation *Φ*$\sqrt{\gamma_{s}}$ vs. *cos φ* (**a**), and as the solid surface energy (γ_s_) as a function of *cos φ* (**b**); only R^2^ ≥ 0.9 values are shown.

Wetting is the property that determines a liquid phase’s ability to retain contact with a solid surface. Wetting is based on intermolecular interactions that can lead to adhesivity (liquid-to-surface) and cohesivity (liquid-to-liquid). It is based on dispersed and polar solid components that can integrate with dispersed and polar liquid phase components [60]. However, different solvents with the same ST value may have different values of polar and dispersed contributions and, consequently, exhibit different wetting tendencies. Therefore, the relationships between the different components between both phases provide insights that help explain the wetting results obtained for the developed spin-coating films. In Figure 5a, the relation between cos *φ* and *Φ*$\sqrt{\gamma_{s}}$ is presented, while in Figure 5b, the *cos φ*, as a function of solid surface energy, is demonstrated.

In Figure 5a, the plotted values of *cos φ* vs. *Φ*$\sqrt{\gamma_{s}}$ are presented. The R-squared values explain the linear dependency between *Φ*$\sqrt{\gamma_{s}}$ and *cos φ*; high correlation in the case of DMSO (R^2^ = 0.98) and large correlation in the case of phi 6 (R^2^ = 0.93) when used as wetting solutions could be observed. The high strength of relationship indicates that since both solutions expressed (very) low polarity, i.e., *P_l_*_(DMSO)_ = 0.18 and *P_l_*_(phi 6)_ = 0.29, and solid glass slides coated with films A, B, and AB also showed very low polarity (dispersed part dominating), one would thus assume that only the dispersed components of solution and solid samples took part in interactions when they came into contact. So, it can be assumed that physical and hydrophobic interactions are the driving force for wettability in DMSO and phi6 dispersions.

**Figure 6:**
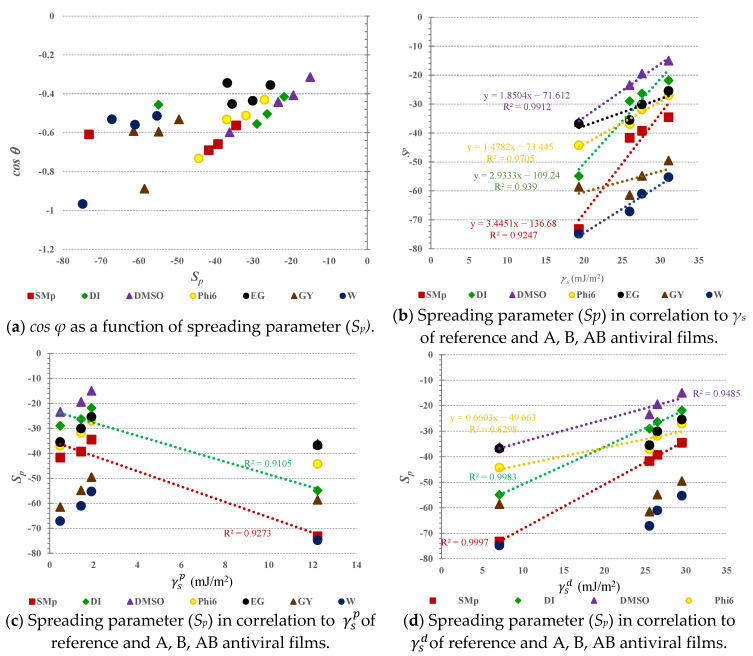
Plots of spreading parameter (*S_p_*) in relationships with *cos φ* (**a**), with total surface energy (*γ_s_*) (**b**), and with corresponding polar ($\gamma_{s}^{p}$) (**c**) and dispersive components ($\gamma_{s}^{d}$ ) (**d**) for A, B, AB antiviral films (note: only significant R-squared results are listed).

In Figure 5b, *cos φ* as a function of $\gamma_{s}$ is shown, indicating an almost perfect linear relationship, in addition to the DMSO and phi6 solutions, and also for GY. This means that when *cos φ* increases, the overall SFE of the solid samples also increases, while the linear correlation is not shown by all of the used test liquid forms. In this sense, the wetting could not be explained total SFE alone. The wetting outcomes due to solid–liquid interactions, where the surface energy of the solid and SFT of the liquid and both their corresponding dispersive and polar components, play a role. The latter is true for DMSO, phi6, and GY solutions, since their dispersive components (i.e.$,\gamma_{l\left(DMSO\right)}^{d}=36$; $\gamma_{l(Phi6)}^{d}=37$; $\gamma_{l(GY)}^{d}=34$) contribute to SFT in surplus compared to polar component (i.e., $\gamma_{l(DMSO)}^{p}=8$; $\gamma_{l(Phi6)}^{p}=15$; $\gamma_{l(GY)}^{p}=30$) donation. In addition, interfacial function *Φ* vs. *cos φ* showed perfect linear association (R^2^ = 1) only when using complete wetting, where *cos φ* equalled 1 and thus *Φ* reached its maximum. The spreading (*S_p_*), as well as the parameter-defining interactions between the solid and liquid, are presented in Figure 6, i.e., the correlation with *cos φ* (Figure 6a), with solid surface energy (Figure 6b), and its polar (Figure 6c) and dispersive contributions (Figure 6d) to the SFE, taking into account all samples including the reference.

In Figure 6a, the regression analysis is presented, allowing for examination of the relation between the independent variable as the spreading coefficient (*S_p_*) and dependent variable, i.e., *cos φ*. Graphical plots evidence that there are no good linear corelations, since all used liquid forms the R-square (not shown) amounted to not being higher than 0.7. The latter indicates that, regardless of the liquid forms used, complete wetting did not occur, since the results of *cos φ* remained under 1, as well as the results of *S_p_* being significantly below zero (note that complete wetting occurs when *cos φ* = 1; thus, *S_p_* > 0).

In Figure 6b, the relation between *γ_s_* plotted as an independent explanatory variable and the spreading parameter as a dependent variable is displayed. The results showed that the total surface energy of the solid samples is the factor influencing the spreading, as indicated by R-squared values ≥0.92 using SMb, DI, DMSO, and phi6. In the case of using DMSO and phi6 as test liquids, an almost perfect linear association was observed, since the R^2^ amounted to 0.99 and 0.97. This means that the dispersive component of ST in liquids dominated and were available to interact with solid film samples that also possessed dominating dispersive SFE components. The latter is even more evident in Figure 6d, where the linear dependence between the spreading and the dispersive contributions of the surface free energies of the solid samples are strongly pronounced for solvents that are less polar, as shown even more clearly in Figure 6c.

### 3.3. Surface Properties of Antiviral Polysaccharide Films Affecting Interactions with Model Virus phi6

The interactions between films A, B, and AB and model virus phi6 were determined using two pathways, i.e., wettability and spreading. First, the wettability prospective by CA using Milli-Q ultrapure water (W) was used to define the hydrophilic/hydrophobic potential of the developed films, since it influences the viral persistence. According to the Milli-Q ultrapure water CA results listed in Table 4, it can be seen that all developed films are rather hydrophobic, since the Milli-Q ultrapure water CAs rose to above 87° compared to the reference sample, more pronounced in A and AB samples (see Table 4).

In sample AB, the “component A” had the predominant effect. Formulation A is chitosan, a polysaccharide that consists of glucose units that possess reactive primary amino groups and OH groups. The difference compared to cellulose is only at the C-2 position, having an amino group instead of −OH; thus, one would expect it to have hydrophilic properties, since it possesses functional groups considered polar (i.e., acid, alcohol, amine). To explain the hydrophobic properties, maybe the chitosan molecules themselves had orientated with reactive groups (i.e., polar hydroxyl and amino) into a bulk phase and/or in parallel with the surface, and with its hydrocarbon side (-CH), described as hydrophobic, to the outside (i.e., available on the surface), as already previously predicted. Consequently, the hydrophobic character, preventing the formation of hydrogen bonds with polar Milli-Q ultrapure water as the liquid, occurred, leading to non-wettability [61]. Film B consists of cationised cellulose nanofibrils; thus, one would expect that two factors would synergistically contribute to its hydrophilic nature, i.e., the nano dimension and cellulose composition possessing many OH groups on its backbone. According to these reasons, the rather high CA obtained with Milli-Q ultrapure water is difficult to interpret. One possible explanation would be the orientation of nanofibrils on the surface of film B [62]. Another explanation could also lie in the dual character (hydrophilic/hydrophobic) of cellulose and its main hydrocarbon chain [63] being oriented at the outer part of the film.

The abovementioned interferences could be further explained by interpreting plotted values of *cos φ* as a function of *Φ*$\sqrt{\gamma_{s}}$, as demonstrated in Figure 5a. The very weak negative linear correlation between the solid films and Milli-Q water could be seen, such as the plotted dots forming the line that slants from right to left with R^2^ < 0.5, indicating that relations between the film’s surface energy and Milli-Q water and the interfacial interaction between them tend to “act” in opposite directions.

Furthermore, the linear plot of *cos φ* vs. *Φ*$\sqrt{\gamma_{s}}$ (Figure 5a) demonstrated moderate correlation, taking into account the solid films and model virus phi6 solution. The latter showed that 92% of the dependent variable, i.e., the total surface free energy of the solid and SFT of the model virus phi6 solution, could explain the interfacial interactions between them. An even better relation between the independent and dependent variables could be observed in Figure 5b, since 98% of the interactions between the developed films and model virus phi6 solution could be explained by SFE of films A, B, and AB. The main reason for the higher CA between the films and the phi6 solution is that the dispersive components contribute to the majority of interactions between the two parties. In the films (A, B, AB), the polar character was almost negligible (Figure 3), and in phi6, some polar components were present (about 29%), which may have contributed to the total SFE. However, these polar forces, responsible for good adhesion, are present, albeit to a very small extent.

**Figure 7:**
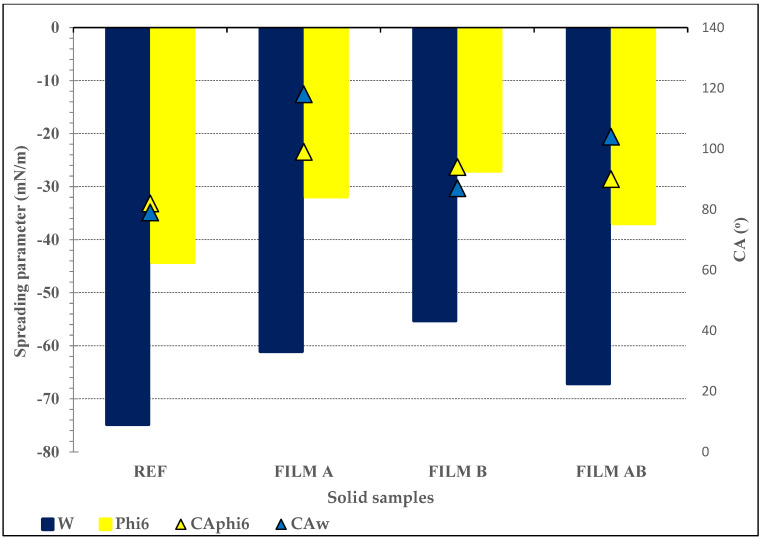
Evaluated spreading parameters (*S_p_*) and measured CAs of Milli-Q ultrapure water (CA_W_) and model virus phi6 solution (CA_phi6_) on reference and spin-coated films A, B, and AB; *S_p_* is presented as bar chart; CA results are presented as triangles.

The second pathway for evaluating the developed films’ wetting potential defined the spreading parameter (*S_p_*) using only Milli-Q ultrapure water (W) and phi6 for emphasis, the hydrophilic/hydrophobic character, and viral transmission potential. In Figure 7, the spreading parameter of Milli-Q ultrapure water (W) and the model virus phi6 solution (phi6), over the reference, and antiviral films A, B, and AB, are presented along with the corresponding CA results obtained.

Figure 7 shows the negative results of the spreading parameter (*S_p_*) by both chosen liquid forms regardless of solid samples used, pointing to the cohesion work between molecules within the same liquid phase (i.e., W and phi6) being greater than the work of adhesion at the interphase between solid samples and the liquid phase. The latter is more emphasized by Milli-Q ultrapure water (W) since the twofold higher (more negative) spreading parameter values (*S_p_*) could be seen in spin-coated A, B, and AB films compared to the phi6 dispersion. The reason is due to Milli Q ultrapure water’s ability to form hydrogen bonds within its own molecules. Both liquid forms showed no potential to be spread on any of the film samples, more pronounced by chitosan-containing film samples (A and AB) since the cohesion energy of chitosan is pH-dependent (cohesion decreased with increasing pH) [64].

It is known that bacteriophage phi6 is composed of three layers; the outer layer is made of phospholipids [65]. The phospholipids’ amphoteric character allows them to orient the hydrophobic tail and the hydrophilic head upon contact with the liquid phase. So, its nature is also dependent on its conformations and structure. In our study, the dispersive part (hydrophobic) dominated in the phi6 dispersion. It may be assumed that among phi6 and polysaccharide films, weak physical van der Waals forces (dispersion forces) and hydrophobic interactions may occur. As we connected this to real materials, i.e., screen-printing of A, B, and AB formulations onto the first PP layer of face masks, as shown in our previous work [34], it may be concluded that they did not show the desired antiviral effect (not shown here, published previously). A possible reason is, therefore, the poor adhesion between the virus and the polysaccharide-modified material surface during testing.

Among all the samples presented, the PP masks coated using formulation A had the highest log kill value at 1.14 [34], which is still not sufficient for effective virus inhibition, and the highest CA was measured using phi6 and W (solvent in phi6 dispersion). The lower CA with phi6 dispersion, as well as the lowest CA with Milli-Q ultrapure water, was shown in formulation B (87°), which did not detect antiviral efficacy (i.e., zero log inhibition). It was seen that antiviral activity is opposite to water wettability (i.e., water CA) and thus hydrophilic character.

The poor wettability and interactions between phi6 and antiviral compounds (A, B, and AB) affected the test results whilst the virus was not in good enough contact with the test fabric to be deactivated by the active coatings. It would be interesting to extend the testing time or improve the fabric’s wettability. Use of plasma treatment on hydrophobic PP microfibers could be one option for providing new polar functional groups for better adhesion of coatings and different orientation of polysaccharides onto its surfaces, i.e., more polar groups may be available on the surface and thus increase the polar part of the material itself in order to be able to react with the polar part of phi6. In this way, the wettability increases and potentially improves the blocking/inhibition of the virus, which is extremely important, by contact killing mechanism of action.

## 4. Conclusions

To clarify why the same formulations, i.e., high molecular weight chitosan (A), quaternised cellulose nanofibrils (B), and their mixture (AB), in different forms (i.e., coated film layer or liquid phase) result in different antiviral performances, as shown in our previous articles [14,34], the present study focused on exploring interaction mechanisms using wettability studies and mathematical models for evaluating surface free energy parameters. The total SFE and associated dispersion and polar contributions were evaluated for selected polysaccharide spin-coated films using comparisons between the most commonly used mathematical models: Fowkes, Owens–Wendt–Rabel–Kealble (OWRK), Wu, and van Oss–Chaudhury–Good (vOGC). Films A (i.e., HMWCh) and AB (i.e., HMWCh + qCNF) had almost the same total SFE values, which were about 30% higher compared to the reference sample, while the quaternised nanocellulose film (B) had the highest SFE (i.e., about 40% higher than the pure glass slide reference sample). The SFE of all samples consisted largely of the contribution from the dispersive components (more than 93%), while the polar contribution was insignificantly low, i.e., ranging from zero for sample AB to 1 mJ/m^2^ for sample A and 2 mJ/m^2^ for sample B. The lowest total SFE was estimated for the reference sample, i.e., 19 mJ/m^2^, where the contribution of the dispersive components to the total SFE was low, i.e., 7 mJ/m^2^, while the polar contribution was the highest (i.e., 12 mJ/m^2^) due to the polar groups present in this material. For film AB, followed by film A and film B, the contribution of the polar component to the total SFE was very small—close to zero. The latter highlights the dominant dispersive component in all samples formed using spin-coating, which, as mentioned above, is mainly due to the orientation of available surface hydrocarbon chains originating from polysaccharide backbones. The Lewis acid-base character of the polar part is related to the ionizing functional groups on the surface, which have a negligible acidic or basic character due to their low availability and presence. The calculated SFE values for the films varied between the different mathematical models depending on the type of test solvent used, but clearly showed the dominant influence of the dispersive components. The latter was also confirmed by the strong correlations between the Wu, vOGC, and OWRK mathematical models. The correlation between the vOGC and OWRK models showed a dependence between all surface free energy components, while the Wu–OWRK and Wu–vOGC relationships showed correlation only for the dispersive and polar components, respectively. The dispersion parameter, as a useful parameter for the measurement of wetting, showed negative results of the dispersion parameter (*S_p_*) for both selected liquid forms, water and phi6, and, regardless of the solid samples used, indicated that the work of cohesion between the molecules within the same liquid phase (i.e., W and phi6) was greater than the work of adhesion at the interface between the solid samples and the liquid phase. It can be concluded that the cohesive forces in the liquid phase are stronger than the work of adhesion at the contact surface. Weak physical van der Waals forces (dispersion forces) and hydrophobic interactions may occur between phi6 and polysaccharide spin-coated films, resulting in poor adhesion between the virus and the material surface. The poor wettability and interactions between phi6 and the antiviral substance affected the test results, while the virus was not in sufficient contact with the test materials to be further deactivated by the active coatings to lose infectivity. In the case of a noncontact mechanism, the dispersive part may be important, as it prevents viral adhesion and thus also helps to reduce cross-infection, but in this case, the use of coatings was not useful or meaningful.

Our study shows that the dispersive and polar components of the two phases involved play an important role in the adhesion of microorganisms. When the polar component dominates, wettability is better, while the polar sides attract each other. In addition, electrostatic interactions can occur between the coating and viruses, allowing the agent to block the activity of the viruses, which is extremely important for the contact killing mechanism that should logically be performed by the active coatings.

Knowledge of the surface properties of materials and the interaction phenomena to which SFE is important, especially those exposed to a higher number of contacts and/or are considered potentially pathogenic surfaces (fomites), is more than desirable for the development of protective materials that not only meet hygiene standards but also contribute to the adequate prevention of viral pandemics.

## Figures and Tables

**Table 1:** The list of spin-coated film sample notations and descriptions.

Spin-Coated Sample Notation	Description of Polysaccharide Film Sample
A	Spin-coated film on glass slide using polymer solution of 2 wt.% HMWCh
B	Spin-coated film on glass slide using polymer solution of 1.5 wt.% qCNF
AB	Spin-coated film on glass slide using polymer solution of HMWCh and qCNF (prepared in mass ratio HMWCh: qCNF = 1:1 (*w*/*w*))

**Table 2:** The SFT and viscosity of polymer formulations used in spin coating and weight and thickness variation of prepared film coatings on a glass cover.

Polymer Formulation Prior Spin Coating			Film (Spin Coated) on Glass Cover	
Formulation	SFT (mN/m)	Viscosity (mPas)	Weight (g)	Thickness (mm)
A	60.46 ± 0.74	4497.8 ± 52.0	0.1834 ± 0.0021	0.2140 ± 0.0200
B	136.96 ± 38.82	7000.0 ± 123.0	0.4068 ± 0.0052	0.2870 ± 0.2501
AB	63.42 ± 0.49	572.3 ± 46.0	0.1141 ± 0.0009	0.1825 ± 0.0331

**Table 3:** SFT, its polar and dispersive components and polarity of test liquids and SM buffer solution and phi6 dispersion, as used in SFE calculation models.

Liquid Phase	SFT and Their Components			Polarity (*P_l_*)
	SFT $\gamma_{l}$ (mN/m)	Polar Component $\gamma_{l}^{p}$ (mN/m)	Dispersive Component $\gamma_{l}^{d}$ (mN/m)	
SMb	60.5 ± 0.0	0.9 ± 0.0	59.6 ± 0.0	0.02
DI	50.8 ± 0.0	1.8 ± 0.0	49.0 ± 0.0	0.04
DMSO	44.0 ±0.0	8.0 ± 0.0	36.0 ± 0.0	0.18
phi6	51.9 ± 0.1	15.0 ± 0.1	36.9 ± 0.1	0.29
EG	48.0 ±0.0	19.0 ± 0.0	29.0 ± 0.0	0.40
GY	64.0 ± 0.0	30.0 ± 0.0	34.0 ± 0.0	0.47
W	72.8 ± 0.8	50.7 ± 0.4	22.1 ± 0.3	0.70

**Table 4:** CAs of test liquids, buffer solution, and viral surrogate dispersion on glass cover sample without and with coated film.

Sample	CA (°)						
Film	SMb	DI	DMSO	phi6	EG	GY	W
REF	109.0 ± 8.9	93.3 ± 6.8	59.4 ± 2.6	82.3 ± 4.7	95.1 ± 4.0	96.3 ± 2.2	78.6 ± 5.1
A	95.5 ± 3.7	66.8 ± 5.8	37.0 ± 1.2	98.9 ± 3.6	67.3 ± 3.8	91.7 ± 2.5	117.7 ± 4.7
B	86.7 ± 4.9	51.4 ± 3.9	33.1 ± 3.9	94.4 ± 5.7	41.6 ± 3.5	78.5 ± 4.4	86.7 ± 5.7
AB	90.3 ± 4.1	65.2 ± 5.1	36.4 ± 2.9	90.1 ± 3.3	65.4 ± 3.4	90.4 ± 3.8	103.6 ± 4.7

## Data Availability

The data presented in this study are available upon request from the corresponding author.
